# Supplementary material for: Sulforaphane and Benzyl Isothiocyanate Suppress Cell Proliferation and Trigger Cell Cycle Arrest, Autophagy, and Apoptosis in Human AML Cell Line
Source: Int J Mol Sci. 2024 Dec 17;25(24):13511. doi: 10.3390/ijms252413511 (PMC11677715; doi:10.3390/ijms252413511)
Supplement: Supplementary file 1 [file ijms-25-13511-s001.zip › ijms-3302380-supplementary.pdf]

International Journal of *Molecular Sciences*

Sulforaphane and Benzyl isothiocyanate Inhibit Cell Proliferation by Inducing Cell Cycle Arrest and Apoptosis in human AML cell lines.

Anna Bertova 1, Szilvia Kontar 1, Martina Ksinanova 1, Alberto Yoldi Vergara 1, Zdena Sulova 1, Albert Breier 1,2* and Denisa Imrichova 1,2*

1 Institute of Molecular Physiology and Genetics, Centre of Biosciences, Slovak Academy of Sciences, Dúbravská cesta 9, 840 05 Bratislava, Slovakia; anna.bertova@savba.sk (A.B.); szilvia.kontar@savba.sk (S.K.); martina.ksinanova@savba.sk (M.K.); umfgyolv@savba.sk (A.Y.V.); zdena.sulova@savba.sk (Z.S.)

2 Institute of Biochemistry and Microbiology, Faculty of Chemical and Food Technology, Slovak University of Technology in Bratislava, Radlinského 9, 812 37 Bratislava, Slovakia

* Correspondence: albert.breier@stuba.sk (A.B.); denisa.imrichova@savba.sk (D.I.)

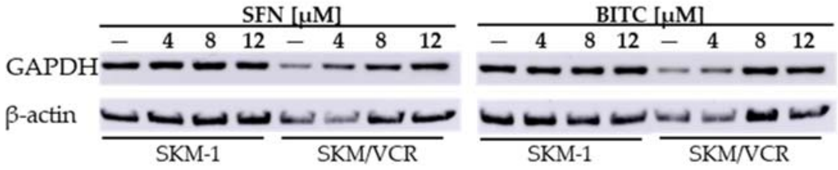

Figure S1. SFN and BITC affect the levels of typically used housekeeping proteins in human AML cell lines SKM-1 and SKM/VCR. Cells were exposed to SFN or BITC (0, 4, 8 and 12 μM) for 12 h. Total protein extracts (60 μg/line) were determined for SDS-PAGE gel electrophoresis and subjected to im-munoblot analysis using anti-GAPDH and anti-β-actin antibodies.

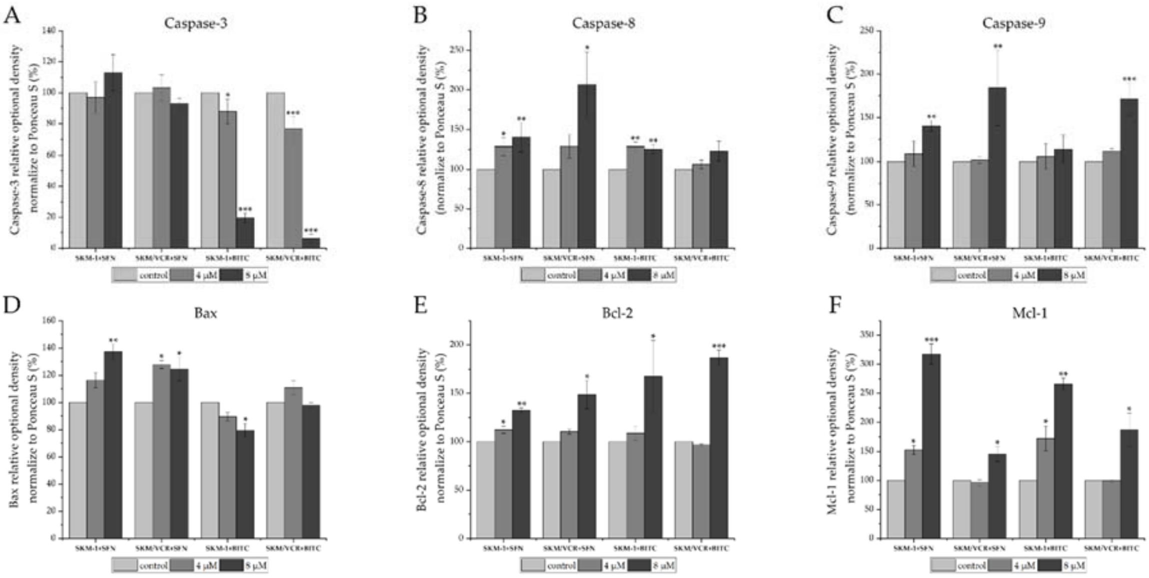

Figure S2. Effect of SFN and BITC treatment on the levels of Bcl-2 family proteins and caspases in SKM-1 and SKM/VCR cell lines. Cells were treated with SFN or BITC (0, 4 and 8 μM) for 12 h. Quantitative analysis of anti-caspase 3, 8 and 9 (A-C) and Bcl-2 family proteins Bax (D), anti-Bcl-2 (E) and Mcl-1 (F). Protein expression was analysed by Western blotting. Red Ponceau staining of total protein signals was used as an internal control. The relative intensity of protein expression was normalised to Ponceau S staining and analysed using ImageJ. Results are expressed as mean ± SD of 3 experiments vs. One-way ANOVA with Tukey's post hoc test (*P < 0.05, **P < 0.01 and ***P < 0.001 vs. control cells) was used to assess the significance of differences in the evaluated parameters.
